# Functional comparison of anti-restriction and anti-methylation activities of ArdA, KlcA, and KlcA_HS_ from *Klebsiella pneumoniae*

**DOI:** 10.3389/fcimb.2022.916547

**Published:** 2022-07-28

**Authors:** Huimin Chen, Shuan Tao, Na Li, Fang Wang, Lei Wang, Yu Tang, Wei Liang

**Affiliations:** ^1^ Medical School of Jiangsu University, Zhenjiang, China; ^2^ Department of Laboratory Medicine, Bengbu Medical College, Bengbu, China; ^3^ Department of Central Laboratory, Lianyungang Second People Hospital, Lianyungang, China; ^4^ School of Biotechnology, Jiangsu University of Science and Technology, Zhenjiang, China; ^5^ Department of Laboratory Medicine, Shanghai East Hospital, Tongji University School of Medicine, Shanghai, China; ^6^ Lianyungang Clinical College of Jiangsu University, Lianyungang, China

**Keywords:** horizontal gene transfer (HGT), restriction–modification (RM) system, *Klebsiella pneumoniae*, anti-restriction protein, ArdA

## Abstract

Anti-restriction proteins are typically encoded by plasmids, conjugative transposons, or phages to improve their chances of entering a new bacterial host with a type I DNA restriction and modification (RM) system. The invading DNA is normally destroyed by the RM system. The anti-restriction proteins ArdA, KlcA, and their homologues are usually encoded on plasmid of carbapenemase-resistant *Klebsiella pneumoniae*. We found that the plasmid sequence and restriction proteins affected horizontal gene transfer, and confirmed the anti-restriction and anti-methylation activities of ArdA and KlcA during transformation and transduction. Among the three anti-restriction proteins, ArdA shows stronger anti-restriction and anti-methylation effects, and KlcA_HS_ was weaker. KlcA shows anti-methylation only during transformation. Understanding the molecular mechanism underlying the clinical dissemination of *K. pneumoniae* and other clinically resistant strains from the perspective of restrictive and anti-restrictive systems will provide basic theoretical support for the prevention and control of multidrug-resistant bacteria, and new strategies for delaying or even controlling the clinical dissemination of resistant strains in the future.

## 1 Introduction

In China, the clinical detection rate of carbapenem-resistant *K. pneumoniae* (CR-KP) has increased sharply in recent years, from 5.5% in 2013 to 10.1% in 2018 (Zhou et al., 2020). Carbapenem resistance in *K. pneumoniae* is determined by the presence of multiple resistance mechanisms in the bacteria, and the production of carbapenemases(such as KPC-2, OXA-48, NDM-1, VIM, etc.) is currently the main mechanism of carbapenem resistance in CR-KP (Fang et al., 2019; Makharita et al., 2020; Zhu et al., 2021). KPC enzymes are the carbapenemases most widely reported throughout the world and the probability of infection and eventual death of patients caused by KPC-producing CR-KP can be as high as 75% (Koraimann and Wagner, 2014). The KPC enzyme in *K. pneumoniae* is usually encoded by the *bla*_KPC_ gene, which is almost always located on a plasmid, and the horizontal transfer of the plasmid between strains is critical for the spread of clinical resistance (Abed et al., 2021). The presence of restriction resistance proteins (ArdA, ArdB, and KlcA) in many important pathogenic species allows foreign mobile genetic elements (plasmids, conjugative transposon, and phages) to evade the type I RM system of the host bacterium (Nordmann et al., 2011).

The *ard* genes (alleviation of restriction of DNA) are found in the leading regions of conjugative plasmids and are among the first to reach the recipient cell after conjugative DNA transfer (Chilley and Wilkins, 1995; Dimitriu et al., 2019; Dimitriu et al., 2020). The *ard* genes allow plasmids to overcome restrictive barriers during the transfer of DNA across bacteria of various species and genera, and encode specialized inhibitors of type I restriction endonucleases (Melkina and Zavilgelsky, 2021).

The anti-restriction protein ArdA belongs to the DNA mimic protein family (protein mimicry of DNA), in which the spatial structures of the proteins imitates the B structure of double-stranded DNA (Chen et al., 2014). This protein mimicry allows an anti-restriction protein to compete with DNA for the binding site of an RM enzyme and thus inhibit the degradation (restriction) and methylation (modification) of the DNA (Zavilgelsky et al., 2008).

*klcA* is one of three genes in the kilC operon, which is one of the four *kil* loci on IncP plasmids (Larsen and Figurski, 1994)and expression of *kil* genes can be lethal to *Escherichia coli* (Serfiotis-Mitsa et al., 2010). Because KlcA and ArdB are so similar, it is probable that KlcA also functions as an anti-restriction protein. KlcA_HS_ has previously been shown to share 43% identity and 66% similarity with KlcA136 encoded on pBP136 (Kamachi et al., 2006) and to have anti-restriction activity that facilitates horizontal gene transfer (HGT) (Liang et al., 2019). However, KlcA from the IncP-1a plasmid RK2 showed no anti-restriction activity against the archetypal type I RM system of *E. coli*, EcoKI (Larsen and Figurski, 1994; Lazdins et al., 2020). The RM system (Fullmer et al., 2019)is the first line of intracellular defense for bacterial immune defense, which can rapidly cleave foreign DNA by recognizing specific DNA sequences and triggering restriction endonuclease REase activity, but DNA escapes cleavage when sites are methylated by MTase. In other words, DNA methylation protects it from the restrictive effect of the RM system, which promotes HGT (Price et al., 2016).

Here, we confirmed that ArdA and KlcA isolated from carbapenemase-resistant *K. pneumoniae* function *in vivo* against the restriction and methylation of the type I RM system. In this experiment, *klcA* genes were obtained from two hospital isolated CR-KP plasmids, *klcA* and *klcA*_HS_. However, contrary to expectations, we found that the KlcA protein was unable to block the normal restriction endonuclease activity of the type I RM enzyme EcoKI, indicating that the KlcA protein does not necessarily function as an anti-restriction protein, which is related to the polymorphism of the *klcA* gene.

## 2 Materials and methods

### 2.1 *Escherichia coli* strains, plasmids, and genes

*Escherichia coli* DH5α (Tiangen, Beijing, China) was used as a general cloning strain, and *E. coli* BL21(DE3) (Invitrogen, Groningen, The Netherlands) as an expression strain. *E. coli* NM1261 (restriction and modification negative; R^−^M^−)^ and *E. coli* NM1049 (*Eco*KI, type IA RM system) were gifts from Professor David T. F. Dryden (EaStChem School of Chemistry, University of Edinburgh, UK) and were used to assess the anti-restriction and anti-modification activities of ArdA, KlcA, and KlcA_HS_ *in vivo*. The genes *ArdA*, *KlcA*, and *KlcA*_HS_, encoding the anti-restriction proteins, were sequenced according to National Center for Biotechnology (NCBI) GenBank database under accession numbers: MW581616.1, base sequence 171,141–171,647; CP088129.1C, base sequence 77784–78212; and KP125892.1, base sequence 6166–6591.

### 2.2 Plasmid construction

The KlcA gene on the plasmid (pHS10842) in carbapenem-resistant *K. pneumoniae* strain isolated from Huashan Hospital (Shanghai, China) is referred to hereafter as ‘KlcA_HS_’. The names ‘ArdA’ and ‘KlcA’ without subscripts refer to the ArdA and KlcA gene on the plasmid (pLYG-1) in a carbapenem-resistant *K. pneumoniae* strain isolated from the Second People’s Hospital of Lianyungang City, Jiangsu Province, China.

To evaluate the anti-restriction activities of ArdA, KlcA, and KlcA_HS_, the *ArdA*, *KlcA*, and *KlcA*_HS_ genes were cloned from *K. pneumoniae* plasmid pLYG-1, pHS10842 with predesigned primers containing the *Nde*I and *Xho*I recognition sequences (**Table 1**). Each gene was then ligated into the ampicillin-resistant expression vector pET20b, at the *Nde*I and *Xho*I sites. DNA sequencing in both directions with T7 and T7T primers confirmed that the gene was inserted in the correct position, without base mutations, as indicated in **Table 1**.

**Table 1:** The underlined part of the primer is the introduced restriction endonuclease recognition site.

PCR name	Sequence (5' − 3')	Tm (°C)	Amplicon size (bp)
ArdA	CCGCTCGAGTCAGTTGCTGAATACATAGCCTT GGAATTCCATATGATGACTTCATCAGTAACCGTT	55	507
KlcA	CCGCTCGAGTTAATCAATGGCACGATAAATA GGAATTCCATATGATGGAAACTATCGAAATCAC	55	429
KlcA_HS_	GGAATTCCATATGATGATGCAAACAGAACTTAA CCGCTCGAGGTCTATTGCGGCCAAGATGG	57	426
T7 T7T	TAATCGACTCACTATAGGG TGCTAGTTATTGCTCAGCGG	55	302
P1	ACGTTTTGCAGCAGCAGTCGCGGGTCAATGCC AGCGCTTCG	55	3566
P2	CGAAGCGCTGGCATTGACCCGCGACTGCTGCT GCAAAACGT	60	519
P3 P4	TCGTATTAATTTCGCGGGATC CACTGATGCCTCCGTGTAAG		

### 2.3 Effect of EcoKI enzyme recognition site on plasmid transformation efficiency

We knocked out an EcoKI enzyme recognition site in pET20b by referring to the method published by Zhang et al (Zhang et al., 2014). Primers were designed using Primer Premier 6.22 software. Primers P1/P2 (**Table 1**) were used to reverse amplify the entire pET20b sequence by high-fidelity PCR Kit (Takara, Dalian, China) along the outer EcoKI enzyme recognition site to obtain a loop-opening plasmid without the EcoKI enzyme recognition site. The PCR products separated by electrophoresis and purified using the E.Z.N.A.^®^ Gel Extraction Kit (Omega Biotek, Norcross, GA, USA). Finally, the linear DNA of the loop-opening plasmid was looped again into the mutant pET20b using the Hieff Clone™ Plus One-Step Cloning Kit (Yeasen, Shanghai, China).

The above mutant pET20b product was transformed into Competent RM-deficient cells, inoculated into Luria-Bertani (LB) broth containing 100 μg/mL ampicillin (AMP), and incubated overnight at 37°C. The plasmids of positive colonies were amplified using P3/P4 primer pairs (**Table 1**), and the products were then confirmed by DNA sequencing, and the plasmids were named “ΔEcoKI pET20b”. Under the same experimental conditions, Competent *E.coli* NM1049 (IA) cells were transformed with ΔEcoKI pET20b and pET20b plasmids at a concentration of 0.02 pmol, and the number of transformants formed by each strain on LB agar plates containing AMP were counted, and the experiments were repeated three times in parallel.

### 2.4 Expression and purification of proteins

The expression construct plasmid of pET20b was transformed into *E. coli* BL21 (DE3) and plated onto LB agar containing 100 μg/mL AMP. Two to three colonies were picked and grown in LB broth medium containing 100 μg/mL AMP antibiotic and shaken at 37°C until the optical density at 600 nm wavelength (OD600) reached 0.5. At this point, a final concentration of 1 mM isopropyl β-D-1-thiogalactoside (IPTG) was added to induce expression of the anti-restriction protein gene. The cells were grown at a low temperature (16°C) for up to 12 h before harvesting. Supernatants were obtained by sonication of *E. coli* BL21 (DE3) broth and centrifuged at 12,000 rpm for 30 min. The supernatant was then purified using a nickel column to obtain the anti-restriction protein. Finally, SDS-PAGE (12% gradient gel) analysis was used to assess the expression of recombinant proteins.

### 2.5 Assessment of ArdA, KlcA, and KlcA_HS_ activities by plasmid transformation

Wild-type pET20b and the recombinant plasmid (pET20b–ArdA, pET20b–KlcA, and pET20b–KlcA_HS_) were transformed into *E.coli* NM1261 (restriction and modification negative; R^-^M^-)^ competent cells with reference to the assessment of the anti-restriction activity of KlcA_HS_ by Liang et al (Liang et al., 2017). The above four unmethylated plasmids of *E.coli* NM1261 were extracted with the TIANprep Mini Plasmid Kit II (Tiangen, Beijing, China) and analyzed with a NanoDrop™ 2000 spectrophotometer (Thermo Fisher Scientific, USA) to quantify the concentrations of the four plasmids. Molecular weights of wild-type pET20b and recombinant plasmids were calculated by number of bases using SnapGene Viewer software (GSL Biotech, snapgene.com).

Equal molar concentrations of wild-type and recombinant plasmids were added to 100 μL of *E. coli* NM1049 (IA) competent cells in their respective volumes, gently mixed and placed on ice for 30 min, and then heat-stimulated for 90 s in a 42°C water bath. The cells were then placed on ice for 2 min, and 900 μL of LB broth was added to obtain a total volume of 1 mL of bacterial broth. After shaking at 37°C for 1 h, 100 μL of the bacterial broth was spread onto LB agar plates containing 100 μg/mL AMP and incubated overnight. The transformed colonies were counted the next day (**Supplementary Figure 1**).

To assess the anti-modification activity of ArdA, KlcA and KlcA_HS_, the TIANprep Mini Plasmid Kit II was used to extract methylated wild-type pET20b and recombinant pET20b-ArdA, pET20b-KlcA and KlcA_HS_ plasmids from *E. coli* NM1049 carrying the type IA RM system. Equimolar concentrations of methylated plasmids pET20b-ArdA, pET20b-KlcA, pET20b-KlcA_HS_ and pET20b were transformed into competent *E. coli* NM1049 (type IA RM system) cells. For detailed procedures, refer to the section on transformation for anti-restriction activity, and finally count *E. coli* NM1049 transformants containing pET20b-ArdA, pET20b-KlcA, pET20b-KlcA_HS_ and pET20b (**Supplementary Figure 2**).

### 2.6 Assessment of *in vivo* activities of ArdA, KlcA, and KlcA_HS_ with phage lambda

We determined the activity of ArdA, KlcA or KlcA_HS_ *in vivo* by comparing the efficiency of phage λ infestation of *E. coli* NM1049 (type IA RM system) strains, as previously described (Garcia et al., 2003). All assays were performed in triplicate and at least 30 phage plaques were counted per plate per experiment.

To compare the anti-restriction activities of the plasmids, *E. coli* NM1049 (type IA RM system) cells containing plasmid expressing the *ArdA*, *KlcA*, or *KlcA*_HS_ gene were infected with equal volumes of phage lysate from *E. coli* NM1261 (R^−^M^−^) cells. The number of phage spots after phage infestation of *E. coli* NM1049 were counted to assess the magnitude of anti-restriction activity of the three anti-restriction proteins. The negative control was *E. coli* NM1049 (IA) containing the vector plasmid pET20b (**Supplementary Figure 3**).

Anti-modification activity was assessed by phage lysates from *E. coli* NM1049 (type IA RM system) containing the gene expressing ArdA, KlcA, or KlcA_HS_ infest with equal volumes of *E. coli* NM1261 (R^−^M^−^) and NM1049 (IA), respectively, and phage spot differences between the two were compared. pET20b was used as the negative control (**Supplementary Figure 4**).

### 2.7 Statistical analysis

The results were analyzed and statistical graphs were drawn with the GraphPad Prism 8 for Windows software program. Data derived from more than two groups were compared with one-way analysis of variance (ANOVA) and a one-way *t* test was used to compare two groups. Differences were considered significant at *P* < 0.05.

## 3 Results

### 3.1 Plasmid construction

**Figure 1:**
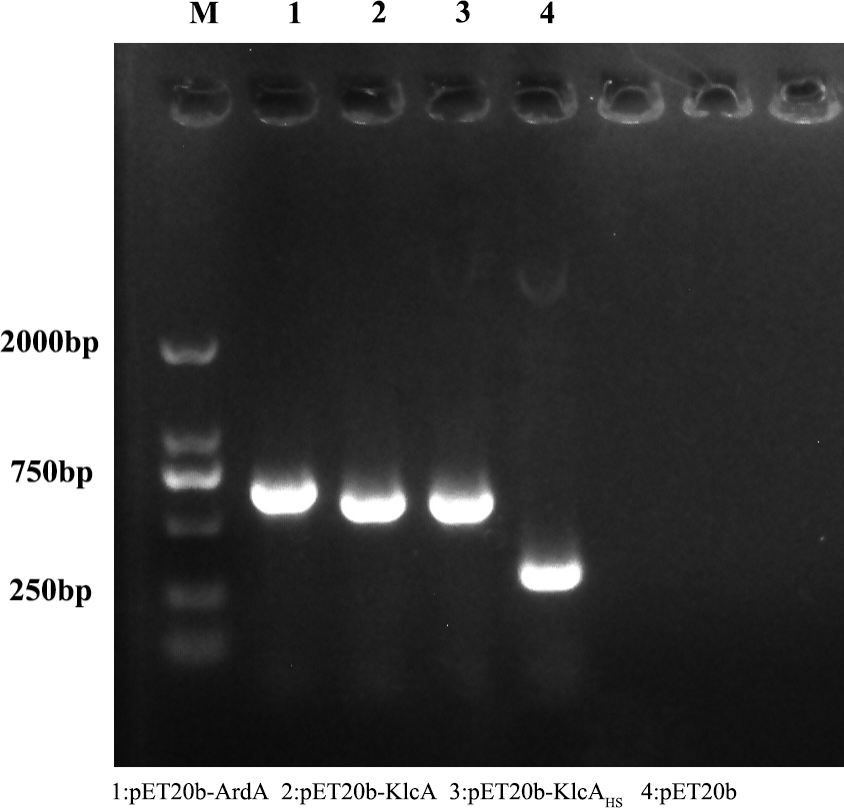
The figure shows the results of agarose gel electrophoresis of the recombinant protein plasmid and the empty plasmid pET20b amplified with T7/T7T primers.

We successfully cloned the *ArdA*, *klcA*, and *klcA*_HS_ genes into the expression vector pET20b and amplified the target fragments with the universal primers T7 and T7T. The correct construction of the recombinant plasmids pET20b–ArdA, pET20b–KlcA, and pET20b–KlcA_HS_ was confirmed with gel electrophoresis (**Figure 1**) and DNA sequencing, and no base mutations were detected.

### 3.2 Protein expression

#### 3.2.1 Expression of ArdA protein

**Figure 2:**
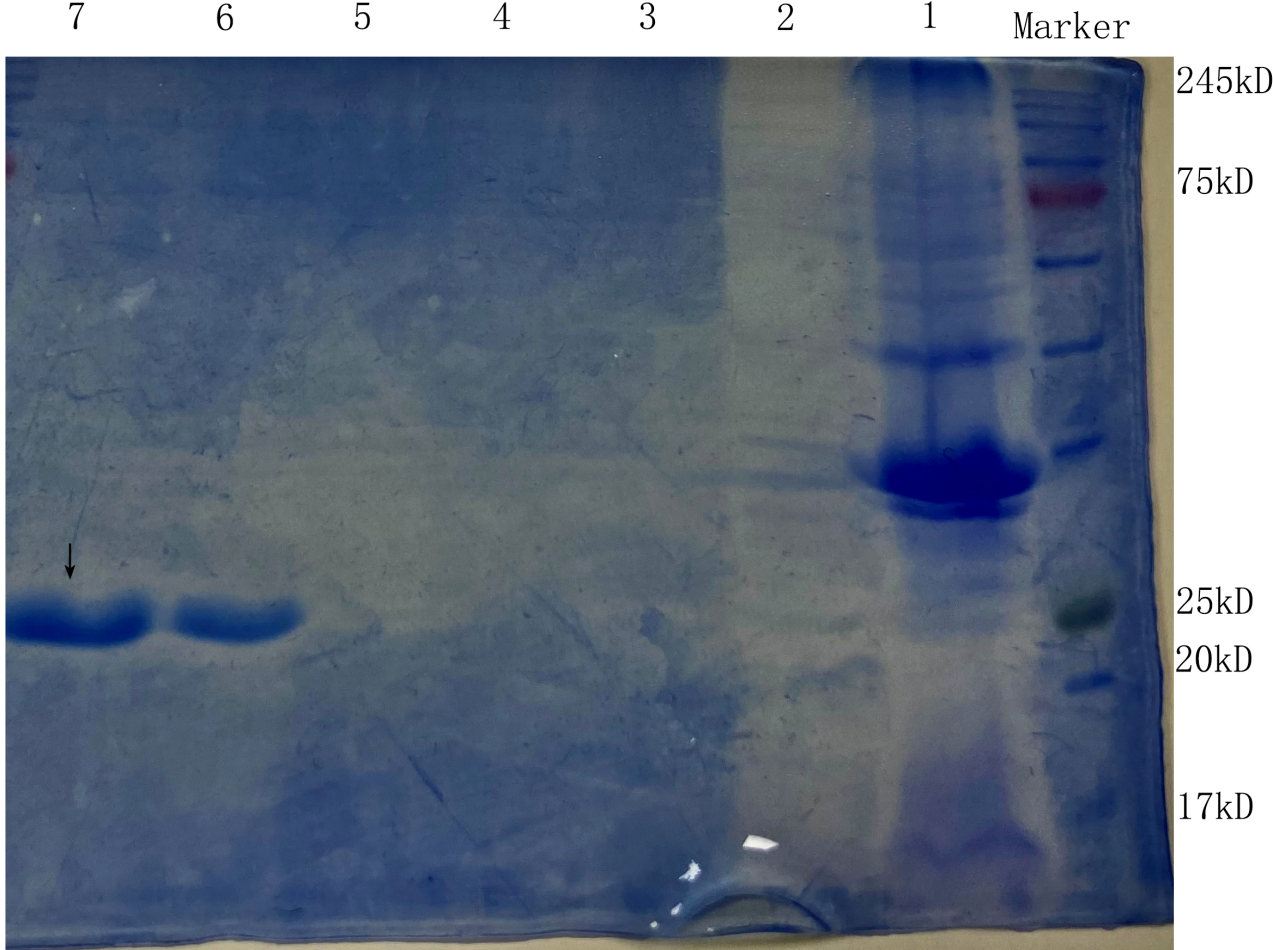
The SDS-PAGE of the anti-restriction protein ArdA. Lanes 1 and 2 are the precipitate and supernatant of *E. coli* BL21 (pET20b-ArdA) obtained by ultrasonic fragmentation without purification, respectively. Lanes 3, 4, 5, 6 and 7 are the target protein ArdA with His tag obtained after purification with 100 mM-500 mM imidazole-eluting nickel columns, located at the positions indicated by arrows.

After *E. coli* BL21 cells containing plasmid pET20b–ArdA were induced with IPTG overnight at 16°C, an aliquot (20 μL) of the unpurified cell lysate and the purified product from the nickel column were removed for SDS-PAGE analysis, and the target protein was eluted off at 400 -500 mM imidazole concentration (**Figure 2**).

#### 3.2.2 Expression of KlcA_HS_ and KlcA protein

**Figure 3:**
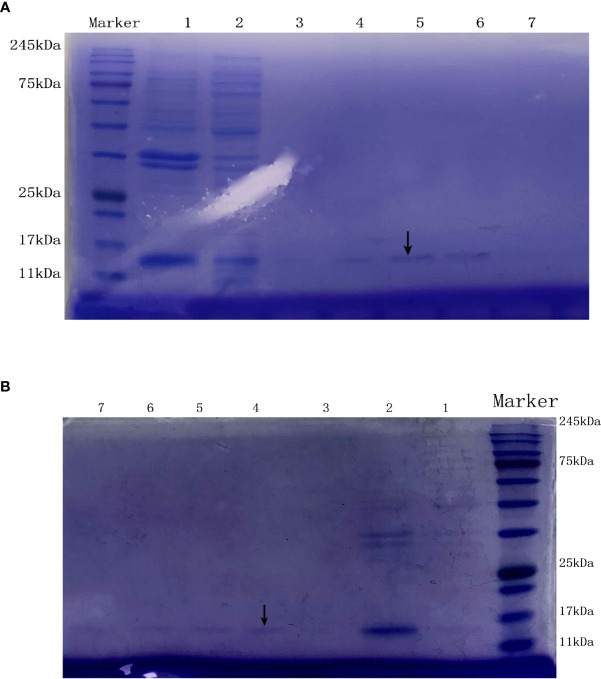
The SDS-PAGE of the anti-restriction protein KlcA **(A)** and KlcA_HS_ **(B)**. Lanes 1 and 2 are the precipitate and supernatant of *E. coli* BL21 (pET20b-KlcA) obtained by ultrasonic fragmentation without purification, respectively. Lanes 3, 4, 5, 6 and 7 are the target protein KlcA with His tag obtained after purification with 100 mM-500 mM imidazole-eluting nickel columns, located at the positions indicated by arrows.

The lysate of *E. coli* BL21 cells containing pET20b–KlcA_HS_ and pET20b–KlcA were loaded onto an affinity chromatography column containing Ni^2+^ and eluted with different concentrations of eluent (50 mM Tris, 50 mM NaCl, 100-500 mM imidazole). The target protein was detected with SDS-PAGE (**Figures 3A, B**).

### 3.3 Restriction enzyme recognition sites

**Figure 4:**
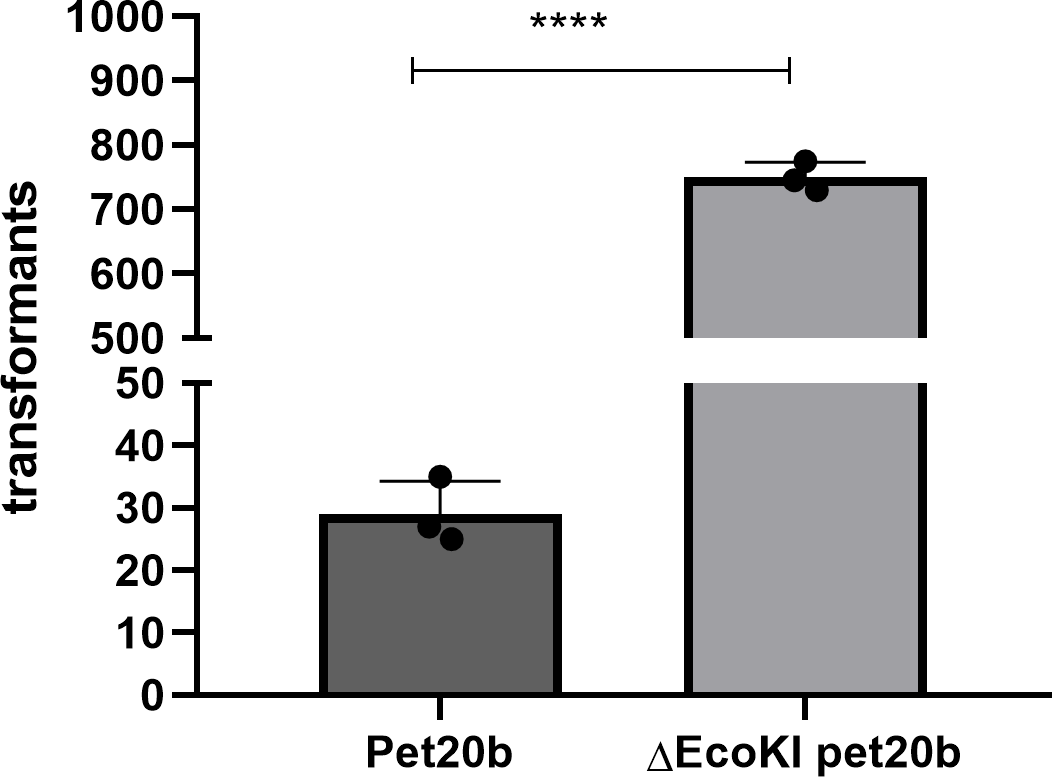
Restriction enzyme recognition site and transformation effificacy. *E. coli* strain (NM1049, EcoKI+) transformed with wild-type plasmid pet20b and Pet20b that lacks one restriction enzyme recognition sites. The number of transformants is indicated. Data are the means ± SD derived from three repeated experiments. ****Signifificantly greater than wild-type; *P* < 0.001.

The restriction enzyme sites in a plasmid significantly influence its transformation efficiency. We found that when the EcoKI recognition site in wild-type pET20b was removed, the transformation efficiency was significantly increased (almost 25-fold) (**Figure 4**).

### 3.4 Anti-restriction and anti-methylation activities of ArdA, KlcA, and KlcA_HS_ *in vivo*

#### 3.4.1 Plasmid transformation

**Figure 5:**
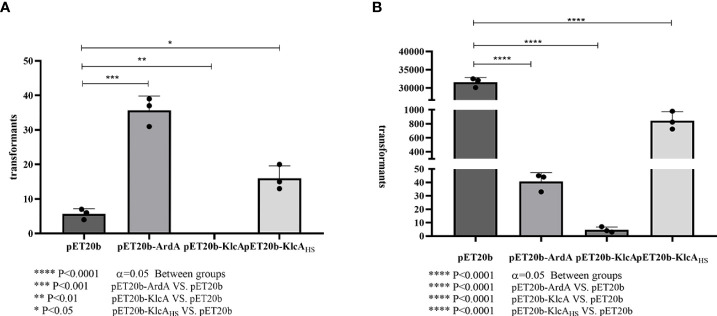
Comparison of anti-restriction and anti-methylation activities of three anti-restriction proteins.The X-axis is the three recombinant proteins and the empty vector pET20b, and the Y-axis is the number of transformants. Transformation efficiencies ratios were calculated by dividing the pET20b-ArdA, pET20b-KlcA, pET20b-KlcA_HS_ transformants with the pET20b transformants. **(A)**: plasmids used were extracted from an R^-^ M ^-^strain. **(B)**: plasmids used were extracted from *E. coli* strain (NM1049, EcoKI+). The assay was repeated in triplicate. Data are the means ± SD.

The anti-restriction activities of ArdA, KlcA, and KlcA_HS_ against type I RM enzymes differed (**Figure 5A**). We used pET20b as the reference plasmid. The anti-restriction proteins ArdA and KlcA_HS_ increased the transformation efficiency of pET20b 6.29-fold and 2.82-fold, respectively, in *E. coli* carrying a type I RM system, whereas KlcA reduced the transformation efficiency. In terms of anti-methylation (**Figure 5B**), the transformation efficiency of a plasmid increases 2.23 × 10^3^-fold after methylation occurs, but the transformation efficiencies of plasmids containing anti-restriction protein ArdA or KlcA_HS_ only increased 4.56- or 210.83-fold, respectively. This suggests that the anti-methylation activities of ArdA and KlcA_HS_ differ in their intensity *in vivo*. KlcA also exerted anti-methylation effects.

#### 3.4.2 Phage transduction

**Figure 6:**
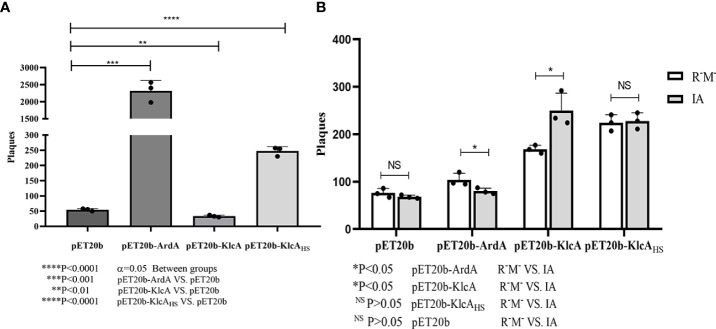
**(A)** X-axis is *E. coli* strain (IA, EcoKI+) containing four kinds of plasmids, Y-axis is the number of plaques obtained by phage infection. **(B)** X-axis shows four kinds of phage from *E. coli* strain (NM1049, EcoKI+) containing four kinds of plasmids, Y-axis shows Λ phage infection of *E. coli* strain (IA, EcoKI+) and R^-^M ^-^strain number of plaques. The assay was repeated in triplicate. Data are the means ± SD.

ArdA, KlcA, and KlcA_HS_ displayed different anti-restriction activities when phage λ was subjected to bacterial type I RM enzymes (**Figure 6A**). We used pET20b as the reference plasmid. The phage-forming efficiency of recipient bacteria expressing the anti-restriction proteins ArdA and KlcA_HS_ increased 42.38-fold and 4.52-fold, respectively, whereas KlcA reduced the transduction efficiency.

However, in contrast to cells expressing no anti-restriction protein (**Figure 6B**), the anti-modification effect of ArdA was generally very weak. In other words, the phage that escaped restriction were easily modified by the methylation activity of the RM system, and KlcA_HS_ had little, if any, inhibitory effect on methylation. Furthermore, KlcA showed no anti-methylation activity (considering the limitations of the experiment), which is not the same as plasmids behaving during transformation.

## Discussion

DNA RM systems are widespread in bacteria and archaea, and function as defense systems to reduce the influx of foreign DNA on mobile genetic elements *via* transduction, transformation, or conjugation (Tock and Dryden, 2005; Picton et al., 2021; Amin et al., 2021). RM systems can be classified into four types, I–IV, depending upon the complexity of their structures and their functions. Type I RM enzymes are the most complex, with both restriction endonuclease and methyltransferase activities (Loenen et al., 2014). These enzymes are composed of three subunits, encoded by the genes *hsdR*, *hsdM*, and *hsdS* (‘hsd’ denotes ‘host specificity of DNA’) (Serfiotis-Mitsa et al., 2010; Roberts et al., 2012). The type I RM systems can cleave exogenous DNA containing specific sequences by recognizing a specific DNA sequence (EcoKI: 5′-AACNNNNNNNGTGC-3′) with the R_2_M_2_S_1_ holoenzyme, whereas the M2S1 enzyme complex has only methylation activity (Loenen et al., 2014). We found that the sequence of the host-borne plasmid and the RM system are key factors affecting HGT, and when one of the two enzyme recognition sites in pET20b was deleted, and it was used to transform an *E. coli* strain containing a type IA RM system, the transformation rate increased approximately 25-fold. This is consistent with the substantial increase in transformation efficiency that occurred after the deletion of the enzyme recognition site in plasmid pHS10842, previously isolated from Huashan Hospital, Shanghai, China (Liang et al., 2017).

Statistics show that natural transformation can occur in about 1% of bacterial species, as transformation involves the uptake and functional establishment of exogenous DNA by the recipient bacteria (Domingues et al., 2012). In many pathogenic bacteria, such as *Staphylococcus* and *Streptococcus*, natural transformation allows them to acquire beneficial traits from distantly related species (Jonas et al., 2001).

We used unmethylated wild-type pET20b to express low levels of ArdA, KlcA, or KlcA_HS_, which usually occur in the absence of induction, and when *E. coli* strains carrying type IA RM systems were transformed with the recombinant plasmids, the transformation rates for ArdA and KlcA_HS_ were significantly increased, whereas that of KlcA was reduced (which is related to the polymorphism of the *KlcA* gene). Furthermore, *in vivo* anti-restriction experiments performed on KlcA expressed from RK2 failed to detect any sign of anti-restriction activity (Larsen and Figurski, 1994), whereas KlcA136 and KlcA_ADE_ showed efficient anti-restriction activity against all four families of type I restriction systems (Serfiotis-Mitsa et al., 2010). To date, the RM system does not appear to be a perfect barrier for foreign DNA, and many plasmids can evade host restriction through a variety of strategies (Tock and Dryden, 2005; Liang et al., 2017).

To date, ArdA and KlcA proteins derived from *E. coli* as well as other bacteria have been assayed *in vivo* to assess their effects on anti-restriction or anti-methylation in four families of the type I RM system (Wilkins, 2002; Thomas et al., 2003; Zavil’gel’skiĭ et al., 2006; Nekrasov et al., 2007; Serfiotis-Mitsa et al., 2010; Liang et al., 2017). When a type I RM system host bacterium contains a plasmid expressing an anti-restriction protein, the magnitude of the anti-restriction protein activity can be tested by determining how many phages successfully infect the bacterium in the case of the host RM system, that is, the number of phage spots on the plate. In our study, both ArdA and KlcA_HS_ proteins, except KlcA, showed anti-restriction activity (>2-fold increase in the number of phage infections relative to the negative control), even though the protein may be expressed at relatively low levels in the absence of induction.

In the presence of ArdA or KlcA, *in vivo* results demonstrated that EcoKI RM enzymes were unable to bind and cleave invading phage DNA, in contrast to *in vitro* results (Serfiotis-Mitsa et al., 2010). ArdA from the conjugative transposon Tn916 (McMahon et al., 2009) has been shown to form elongated structures that bind to the DNA-binding site of the Mtase core of type I RM enzymes, thereby rendering the enzyme incapable of recognizing its DNA target, both *in vivo* and *in vitro* (Chen et al., 2014). In the present study, the anti-methylation effect of ArdA was observed after cells were transformed with plasmid or transfected with phage, and transformation of the methylated plasmid showed this effect more clearly.

Although the mechanism of action of KlcA in inhibiting the RM system remains unclear (Serfiotis-Mitsa et al., 2010; Liang et al., 2017; Balabanov et al., 2019), it has been identified in an increasing number of plasmids and its function varies with genotypic polymorphisms. The expression levels of different *klcA* genes can also vary. We isolated two *klcA* genes from clinically resistant strains, which was not consistent in their ability to inhibit restriction proteins, as observed in this study. These phenomena may reflect the unpredictable differences in their anti-restriction and anti-methylation activities.

The *K. pneumoniae* used here as the source of the ArdA and KlcA proteins, the type I or type II RM system encoded by this bacterial species, and the *E. coli* type I RM system used in this experiment to assess its anti-restrictive activity, are not identical [sequences of type I or II RM systems obtained from the Restriction Enzyme Database [Rebase] (Tock and Dryden, 2005)]. However, given that the ArdA and KlcA proteins examined here were from the same species, and all acted against the type I RM system of *E. coli* (Dimitriu et al., 2019; Melkina and Zavilgelsky, 2021), it seems reasonable to predict that they will also work against the type I RM systems of *K. pneumoniae*, *Enterococcus faecalis, Bacteroides fragilis*, and *Staphylococcus aureus*, or even against any type I RM system.

An interplay between anti-RM systems and RM systems is beneficial for the evolution of bacterial species (McMahon et al., 2009). Despite the observed variations, the fact that ArdA or KlcA blocked restriction to a measurable extent in an unnatural host, even though high levels of protein expression were not induced, suggests that ArdA and KlcA function efficiently as anti-restriction proteins if expressed in their normal hosts and are capable of modulating HGT.

## Data availability statement

The raw data supporting the conclusions of this article will be made available by the authors, without undue reservation.

## Author contributions

CH: wrote the original draft, formal analysis, conceptualization. LW: conceptualization, reviewed and edited the manuscript, supervision. TS, LN: validation, investigation, data curation. WF, WL: validation, investigation. All authors contributed to the article and approved the submitted version.

## Funding

This study was supported by grants from the Natural Science Foundation of Jiangsu Province (BK20191210), the fifth phase of the “333 Project” Scientific Research Project in Jiangsu Province (BRA2019248), the Jiangsu Commission of Health (H2018073), and the Subject of Lianyungang Science and Technology Bureau (SF2015).

## Acknowledgments

The authors would like to thank all the investigators and the staff of the Second People’s Hospital in Lianyungang City, China, for the provision of clinical isolates.

## Conflict of interest

The authors declare that the research was conducted in the absence of any commercial or financial relationships that could be construed as a potential conflict of interest.

## Publisher’s note

All claims expressed in this article are solely those of the authors and do not necessarily represent those of their affiliated organizations, or those of the publisher, the editors and the reviewers. Any product that may be evaluated in this article, or claim that may be made by its manufacturer, is not guaranteed or endorsed by the publisher.
