# Supplementary figures and images for: Functional comparison of anti-restriction and anti-methylation activities of ArdA, KlcA, and KlcAHS from Klebsiella pneumoniae
Source: Front Cell Infect Microbiol. 2022 Jul 28;12:916547. doi: 10.3389/fcimb.2022.916547 (PMC9366191; doi:10.3389/fcimb.2022.916547)

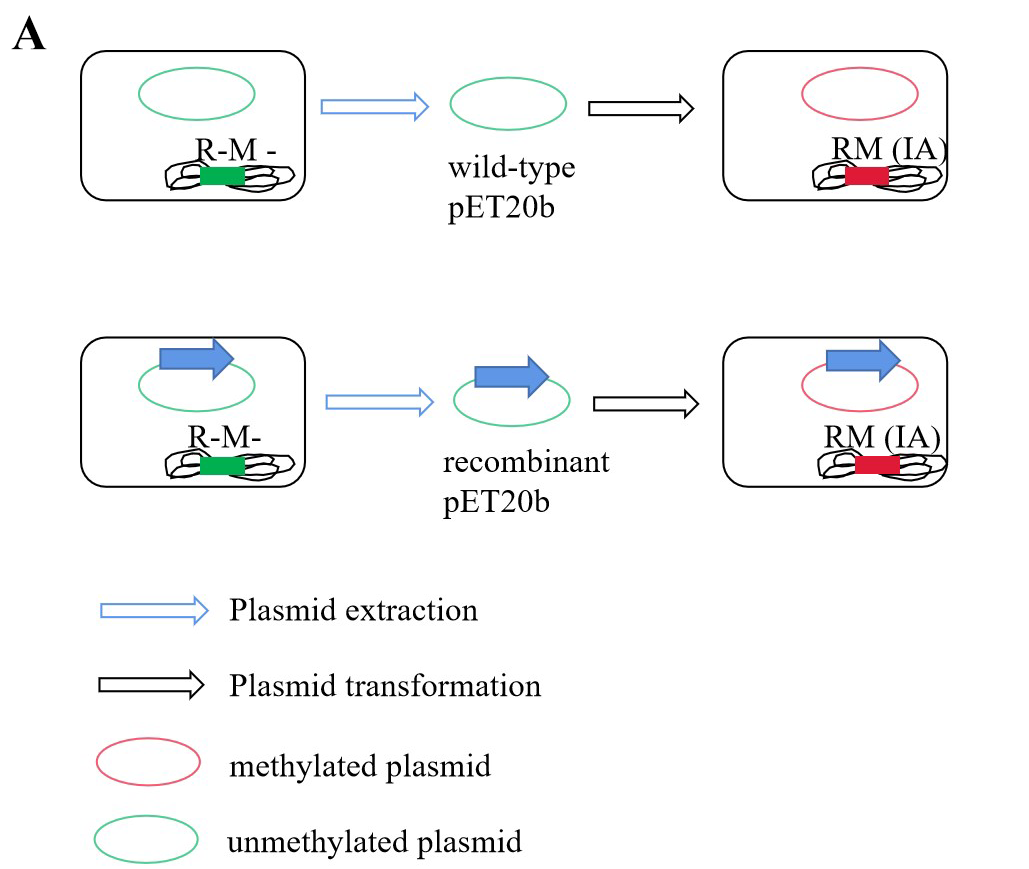

Supplement: Supplementary file 1 [file Image_1.tif]

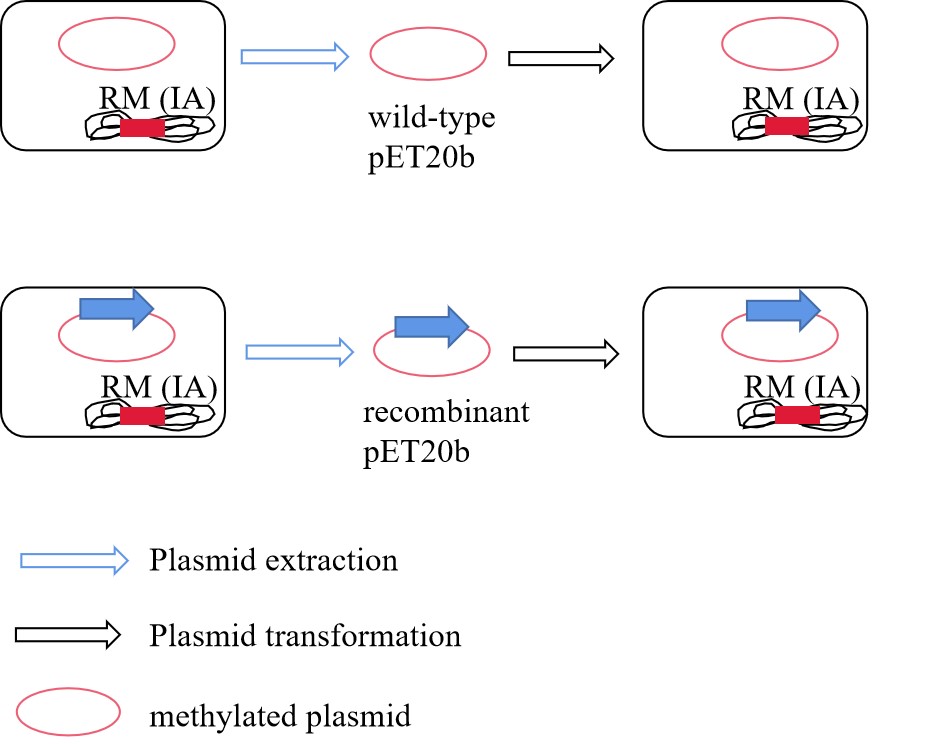

Supplement: Supplementary file 2 [file Image_2.jpg]

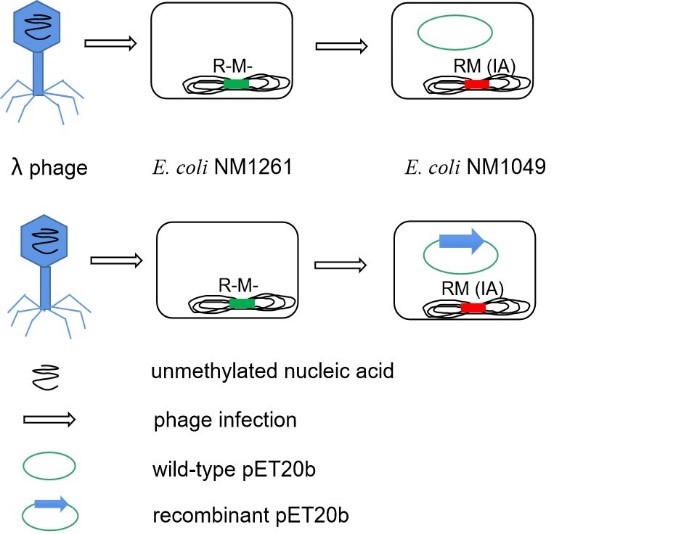

Supplement: Supplementary file 3 [file Image_3.jpg]

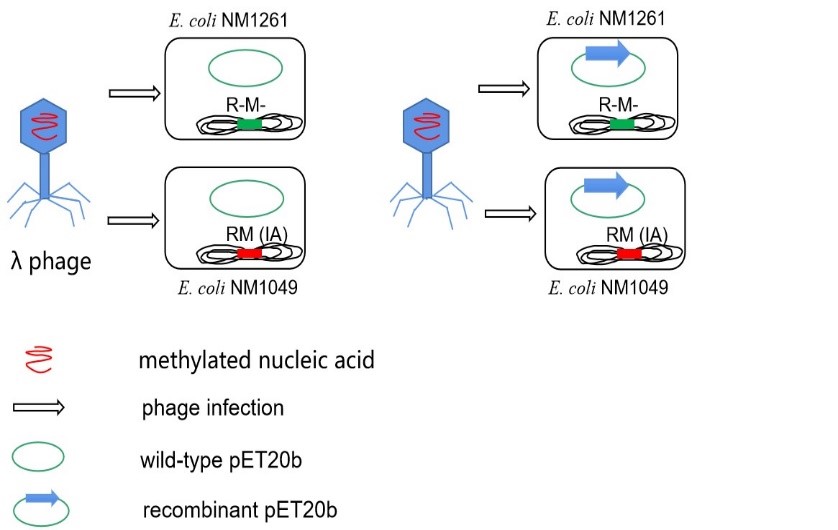

Supplement: Supplementary file 4 [file Image_4.jpg]
